# The effects of initial participation motivations on learning engagement in transition training for future general practitioners in rural China: perceived deterrents as mediator

**DOI:** 10.3402/meo.v21.30998

**Published:** 2016-06-21

**Authors:** Guan-yu Cui, Mei-lin Yao, Xia Zhang, Yan-kui Guo, Hui-min Li, Xiu-ping Yao

**Affiliations:** 1 School of Psychology, Institute of Developmental Psychology, Beijing Normal University, Beijing, China; 2 Department of Psychology, Henan Medical College, Zhengzhou, China; 3 Department of Nursing, Henan Medical College, Zhengzhou, China; 4 Department of Continuing Education, Henan Medical College, Zhengzhou, China

**Keywords:** transition training, general practice, initial participation motivations, perceived deterrents in training, learning engagement

## Abstract

**Background:**

For the shortage of high-quality general practitioners (GPs) in China's rural areas, Chinese government has taken steps to encourage rural specialists to participate in transition training for future GPs. Specialists’ initial participation motivations and their perceived deterrents during training may play important roles for their learning engagement in the transition training. This study aimed at revealing the relationships among the variables of initial participation motivations, perceived deterrents in training, and learning engagement.

**Methods:**

A questionnaire survey was used in this study. A total of 156 rural specialists who participated in transition training for future GPs filled out the questionnaire, which consisted of the measurements of initial participation motivations, perceived deterrents, and learning engagement in training. The data about specialists’ demographic variables were collected at the same time.

**Results:**

The variance of initial escape/stimulations motivation significantly predicted the variance of learning engagement through the full mediating role of perceived deterrents in training. In addition, initial educational preparation motivations predicted the variance of learning engagement directly.

**Conclusions:**

Specialists’ initial participation motivations and perceived deterrents in training played important roles for learning engagement in the transition training.

For the establishment and carrying out of general practice primary care system in mainland China (1), the shortage of high-quality general practitioners (GPs) has become an emergent problem, particularly in China's rural areas (2–6). To solve the problem of GPs shortage in rural areas, Chinese government has taken steps to encourage existing rural specialists to participate in a 1-year transition training program for future GPs (7, 8).

## Training programs of rural GPs in China

There are three training programs for future GPs in mainland China, 5+3 program, 3+2 program, and the program of specialists’ transition training in rural areas, respectively.

The 5+3 program for GPs has been carried out in mainland China for many years. The GPs candidatures should receive 5 years undergraduate education on clinical medicine or traditional Chinese medicine and then engage in 3 years of residency training. However, because of the huge shortage of future GPs and significant urban–rural differences in mainland China, most of the GPs who finished the 5+3 program would like to work in urban areas rather than in rural areas. In addition, unattractive salaries as well as relative low social position compared to specialists may increase the outflow of GPs (1–5).

To increase the amount of future GPs in underdeveloped rural areas rapidly, Chinese government has proposed 3+2 directionally trained program. The GP candidatures in this program should receive 3 years undergraduate education on clinical medicine or traditional Chinese medicine and 2 years residency training. After they receive their degree, the GPs should choose their work places in rural areas. Unfortunately, this program has not yet achieved the expected goals (9).

Therefore, an alternative program has been formulated by the Chinese government. It is the directionally trained program, but the main purpose of this program is to provide an intensive re-training for qualified medical practitioners or assistant medical practitioner specialists in rural areas. As one of the most important and popular training programs in mainland China, this transition training has been carrying out on the national scale (7, 8). Compared with the 5+3 training program and other more rigorous programs for training GPs in the UK and US (10–12), the transition training program in rural China may be a kind of speeded-up solution. The contents of the training include theoretical training (not less than 1 month), clinical skill training (not less than 9 months), and primary care practice (not less than 2 months). In general, theoretical training includes two modules, medical humanistic curriculum and professional core course. The module of medical humanistic curriculum includes Medical Ethics, Medical Psychology, Medicine and Society, etc. The module of professional core course includes General Practice Outline, Basic Medicine and Rehabilitation Medicine, Prevention, Healthcare and Epidemic Medicine, etc. (8). After completing the whole training and passing the examinations, they can get the certificate of specialists’ transition to GPs and register as a GP or assistant GP in their local places (13). At the same time, their wage and treatment may be improved to some extent.

Compared with routine continuing medical education, the transition training program is to provide a new accreditation of GPs for qualified specialists rather than to update the previous accreditation. Therefore, the transition training program may provide more relevant courses or modules and need full-time intensive re-training for about 12 months. In addition, the specialists could make their own decisions on if they need to participate in the transition training program.

As the transition training program is just starting throughout the country, there are many questions to be answered. What kinds of motivations do participants have to attend the training? Which factors would influence participants’ engagement and effects of training? It is necessary to investigate these issues in order to increase the efficiency of the transition training.

## Initial participation motivations, deterrents, and learning engagement

Research has found that there are some important factors that influence participants’ engagement in training as well as training outcomes. Boshier (1971) argued that an individual learner's reasons for participation were the important starting points for his or her study (14–17). Studies also found that the reasons for participation in continuing medical education influenced health workers’ clinical performance (18, 19). Similarly, specialists’ reasons for training participation may affect the quality of future GP service (7). These findings suggest that initial participation motivations play important roles for both process and outcomes of learning or training.

In addition, some researchers found that perceived barriers during participation may affect the training outcomes (20–22). For adult learners, there may exist more or less difficulties and challenges throughout their participation in training due to internal or external factors such as time constraints, family problems, etc. Research found that perceived difficulties or barriers during participation led to changing their roles to rural GPs (23). Based on the assumption of the control-value theory of achievement emotions, learning goals of learners can affect participants’ perceived task value and emotions in learning, and then affect their learning engagement and achievements (24). That means participants’ motivations, as well as perceived barriers about participating in training, are important predictors for learning engagement. Furthermore, trainees’ engagement in training may be the essential factor for the effectiveness of training and participants’ future performance (25–28).

The motivations, perceived deterrents and learning engagement may be the most influential factors for training outcomes. However, there were few studies paying attention to the learning engagement in continuing medical education and specialists’ re-training for future GPs. Previous research focused mainly on the predictive effect of current participation motivations on future participation behaviors, while minimal research investigated the effects of initial participation motivations and perceived deterrents in training on learning engagement (25–29).

This study aimed to reveal the comprehensive relationships among initial participation motivations, perceived deterrents, and learning engagement in transition training for future GPs. It is important to investigate the status of the rural GPs’ training effects in mainland China. The research results may reveal the mechanism of learning engagement and have implications for GPs recruiting, effective training and policymaking in other developing countries.

## Methods

### Participants and procedures

In May 2014, rural specialists (*n*=156) who participated in transition training program for future GPs in Henan Province were invited to complete questionnaires, which included contents such as demographic information, initial participation motivations, perceived deterrents, and learning engagement in training. Participants came from rural areas of eight prefecture-level cities in Henan Province of mainland China and all of them were voluntary to participate in the survey.

### Instruments

#### Rationality of choosing instruments

There were three scales used in present research. Boshier's Education Participation Scale (EPS) is a well-known and widely used instrument in measuring participation motivations in adult education (14–19). The Deterrence to Participation Scale (DPS-G), developed by Darkenwald et al., was often used to investigate perceived barriers to participation of training (20, 21). The Utrecht Work Engagement Scale for Students (UWES-S) was one of the most widely used instruments for assessing learning engagement (29). All of the three scales have favorable reliabilities and validities and are often used for investigating adult learners participating in various training programs. Since the transition training program for future rural GPs is one of the types of adult education, it is reasonable to apply the above-mentioned instruments.

*Initial participation motivations*. Based on the Boshier's EPS (14–17, 30), the present research made a confirmatory factor analysis (CFA) and formed a revised EPS with 20-item, 6-dimension construct. The revised EPS had a good validity (CFI=0.923, TLI=904, RMSEA=0.08) and could be used to assess initial participation motivations in this research (31). Participants rated the extent to which each item best described their reasons for participating in transition training for future rural GPs. A 5-point Likert scale, containing options that extend from 1 (strongly disagree) to 5 (strongly agree), was used to score each item. In addition to six subscales, there was one single-item (‘To satisfy an inquiring mind’) to represent the subscale *Cognitive Interest*. Six subscales are *Social Relations* (four items), *External Expectations* (three items), *Social Welfare* (three items), *Professional Advancement* (three items), *Escape/Stimulation* (three items) and *Educational Preparation* (three items) respectively. *Social relations* stated the need for making new friends, promoting personal association and participating in group activities. *External Expectations* stated the motives to follow others’ instructions, suggestions, and/or requirements. *Social Welfare* stated the motives to devote oneself to community service. *Professional Advancement* stated the needs for higher status in occupation development. *Escape/Stimulation* reflected the need to escape from a boring environment or the incentives to seek out stimulation. *Educational Preparation* reflected the motives to update educational background and improve competences. Sample items include ‘To make new friends’ and ‘To carry out the commendations of some authority’. In this study, the reliability coefficient alpha of the full scale was 0.90.

*Perceived deterrents in training*. DPS-G, designed first by Darkenwald and Valentine, was used to assess perceived barriers to participation (20, 21). It is a 34-item, self-report instrument (21). Four items which were not appropriate for Chinese conditions (e.g., ‘Because the course was offered in an unsafe area’) were deleted and 30 items were used. Six subscales of the scale were *Lack of Confidence* (eight items), *Lack of Course Relevance* (six items), *Time Constraints* (five items), *Low Personal Priority* (five items), *Cost* (three items), and *Personal Problems* (three items), respectively. *Lack of Confidence* stated the low confidence in one's learning ability and lacking of participation encouragement from significant others. *Lack of Course Relevance* stated the course lacking useful or practical contents and activities. *Time Constraints* stated the time conflicts for regular participation. *Low Personal Priority* stated the relative low worthiness of training compared with other activities. *Cost* reflected the lack of financial assistance or reimbursement. *Personal Problems* stated the family or personal health problems. Sample items include ‘Because I was not confident of my learning ability’ and ‘Because the available course did not seem useful or practical’. Participants rated the extent to which each item best described their perceived deterrents in training. A 5-point Likert scale containing options that extend from 1 (strongly disagree) to 5 (strongly agree) was used to score each item. The DPS-G demonstrated a good internal reliability and validity (20, 21). The reliability coefficient alpha of the full-scale in the present study was 0.95.

*Learning engagement in training*. The UWES-S, a 14-item self-report instrument, was used to assess learning engagement (25). Subscales of the scale are *Vigor* (five items), *Dedication* (five items), and *Absorption* (four items). *Vigor* stated the active bodily or mental strength or energy during learning. *Dedication* stated enthusiastic, full of meaning, purpose, and inspiration for learning. *Absorption* reflected the status of complete attention or preoccupation during learning. Sample items include ‘When I study, I feel like I am bursting with energy’ and ‘I find my studies to be full of meaning and purpose’. A 7-point Likert scale containing options that extend from 0 (never) to 6 (always) was used to score each item. Scores ranged from 0 to 84, where a higher score represented higher learning engagement. The reliability coefficient alpha of the full scale in the present study was 0.97.

Data analysis was conducted with PASW statistics for Windows (Version 18, IBM Corp., Armonk, NY) and Mplus software (Version 7.11, Muthén & Muthén, 1998–2013); the level of significance used in this research was *p*<0.05.

## Results

### Demographic characteristics

Table 1 describes the participants’ demographic information.

**Table 1:** Demographic information (*n*=156)

Demographic	*n* (%)
Sex	
Female	86 (55.1)
Age	
20–29	48 (30.8)
30–39	66 (42.3)
40–49	37 (23.7)
50+	5 (3.2)
Years since completed residency	
1–5	44 (28.2)
6–10	22 (14.1)
11–15	32 (20.5)
16–20	28 (17.9)
21–30	27 (17.3)
30+	3 (1.9)
Education level	
Medical secondary school	34 (21.8)
Medical college	94 (60.3)
Medical university	28 (17.9)
Job title	
Medical assistant	81 (51.9)
Physician	54 (34.6)
Physician in charge	16 (10.3)
Associate chief physician	5 (3.2)
Departments	
Internal Medicine	70 (44.9)
Department of Surgery	22 (14.1)
Gynecology	32 (20.5)
Pediatrics	5 (3.2)
ENT	7 (4.5)
Others	20 (12.8)

### Preliminary analysis

Table 2 presents the descriptive statistics of the scales used in this research. The mean, SD (standard deviation), and rank were included. Table 3 presents the intercorrelation coefficients among the variables.

**Table 2:** Descriptive statistics of scales

Scales	Subscales	Mean±SD (*n*=156)	Rank
EPS – revised			
	Professional Advancement	4.33±0.84	1
	Educational Preparation	4.32±0.92	2
	Cognitive Interest	4.17±1.15	3
	Social Welfare	4.02±0.94	4
	Social Relationships	3.73±1.19	5
	External Expectations	3.46±1.16	6
	Escape/Stimulation	3.22±1.11	7
	Total	3.86±0.72	
DPS-G – revised			
	Cost	2.98±1.39	1
	Time Constraints	2.50±1.08	2
	Lack of Course Relevance	2.42±1.17	3
	Personal Problems	2.29±1.13	4
	Low Personal Priority	2.12±0.97	5
	Lack of Confidence	2.12±1.03	6
	Total	2.35±0.88	
UWES-S			
	Dedication	4.81±1.18	1
	Absorption	4.77±1.15	2
	Vigor	4.58±1.16	3
	Total	4.72±1.14	

SD, standard deviation.

**Table 3:** Correlations of the variables

Variables	1	2	3	4	5	6	7	8	9
1. Social relations	1								
2. External expectations	0.56**	1							
3. Social welfare	0.56**	0.51**	1						
4. Professional advancement	0.31**	0.29**	0.64**	1					
5. Escape/stimulation	0.34**	0.44**	0.44**	0.27**					
6. Educational preparation	0.18*	0.13	0.51**	0.68**	0.35**	1			
7. Cognitive interest	0.07	0.03	0.33**	0.38**	0.28**	0.61**	1		
8. Perceived deterrents	−0.16	−0.04	−0.17*	−0.25**	0.16*	−0.18*	−0.13	1	
9. Learning engagement	0.13	0.17*	0.27**	0.38**	0.14	0.45**	0.34**	−0.40**	1

* *p*<0.05

** *p*<0.01

*** *p*<0.001.

### Path analysis

**Fig. 1:**
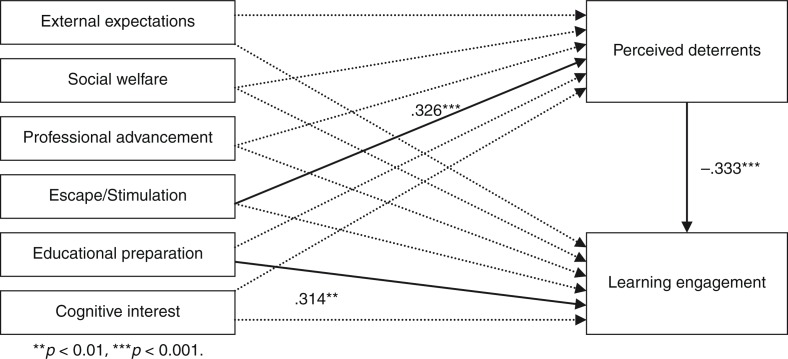
Model of significant (*p*<0.05) relationship among initial participation motivations, perceived deterrents, and learning engagement (*N*=156). Solid line represents the significant pathway, dotted line represents the non-significant pathway (*p*>0.05), and the parameters were significant standard regression coefficients (*p*<0.05).

Figure 1 shows the causal connections between sets of variables, where the solid line represents the significant pathway (*p*<0.05), dotted line represents the non-significant pathway (*p*>0.05), and the parameters were significant standard regression coefficients. A model established revealed the predictive roles of variables such as external expectations, social welfare, professional advancement, escape/stimulation, educational preparation, cognitive interest, and perceived deterrents on learning engagement, while initial social relation motivations could not predict perceived deterrents and learning engagement significantly (*p*>0.05).

As shown in Fig. 1, perceived deterrents in training fully mediated the prediction of initial escape/stimulation motivations on learning engagement in training (*p*<0.001) and initial education preparation motivations predicted learning engagement in training directly (*p*<0.01). The initial motives of external expectations, social welfare, professional advancement, and cognitive interest could not predict perceived deterrents and learning engagement significantly (*p*>0.05).

## Discussion

To facilitate the efficacy of China's current training for future rural GPs, the present research investigated specialists’ initial participation motivations and perceived deterrents in transition training, and their predictive effects on learning engagement in the training. The findings may have implications for improving GPs’ recruiting and training and could provide guidelines for researchers, educators, human resources managers, administrators, and policymakers alike.

### The effects of initial participation motivations on learning engagement

In general, initial motivations were the core predictors on engagement (32–34). As shown in the results of path analysis (see Fig. 1), two kinds of initial participation motivations, that is, escape/stimulation and education preparation, were the significant predictors for specialists’ learning engagement. In particular, the specialists with initial escape/stimulation motivations perceived more deterrents in the transition training and had a lower level learning engagement in the training. On the contrary, the specialists with educational preparation motivations had a higher level of learning engagement as well as better training effects relatively. The results aligned with the explanation and findings in Fujita-Starck's research (35), which investigated ‘why adults participate and how their motivations may vary within population subgroups’. Compared to their initial participation motivations of social relationships, external expectations, social welfare, and professional advancement motivations, education preparation motivations may be one of the most adaptive motivations, while escape/stimulation motivations may be the most maladaptive one.

According to self-determination theory (36, 37), the initial education preparation motivations was the type of autonomous and intrinsic motivation which is related to positive outcomes such as learning engagement in training. Based on Gillet et al.'s (38) study, we found that autonomous motives could predict participants’ positive outcomes significantly. For specialists who participated in the transition training, one of the core goals was to prepare for their new roles as GPs. However, different initial participation motivations may lead to different learning outcomes through different ways. According to the achievement goals theory (39) and recent empirical research findings (40), the participants with intrinsic motivations were more likely to find productive methods or strategies to solve problems, experience positive feelings, and persist in learning activities. On the contrary, the participants with extrinsic motivations were more likely need to get immediate rewards and withdraw or give up their efforts when facing challenges. The present research found similar results. The participants with education preparation motivations may experience more enjoyment and less boredom in learning, which may affect their learning engagement strongly. The participants with escape/stimulation motivation may perceive more deterrents, experience more negative feelings, and engage less in learning.

### The full mediating role of perceived deterrents in training

The results showed that specialists’ initial escape/stimulation motivations affected their learning engagement significantly through the full mediation role of perceived deterrents in training. The specialists with initial escape/stimulation motivations might engage little in learning when they perceive high level of deterrents in training. These findings further confirmed the assumption of the control-value theory of achievement emotions, which suggested that learning goals can affect perceived task value and emotions in learning, and then affect learning engagement and achievement ultimately (24). Additionally, these findings further confirmed previous results that avoidance motivations may be more likely to hurt learning motivations and engagement than approach motivations do (41–47).

Since initial participation motivations and perceived deterrents in training affect learning engagement significantly, it is necessary to find effective methods to change participants’ initial motivations and reduce their perceived deterrents. In fact, it is difficult and unrealistic to increase government investment or extend training time in rural areas of developing countries. In addition, a research on changing motivations of GPs in France showed that increasing extrinsic motivations through policies may generate potential side effects (48). It implied that initial participation motivations could be changed but the corresponding effects may vary with different conditions. How to decrease rural specialists’ perceived deterrents in training through changing their initial inappropriate participation motivations (i.e., escape/stimulation motivations) may need further investigation. Fortunately, there are relative theories and practices on motivation stimulation, which could provide guidelines and strategies for improving rural specialists’ initial participation motivations and changing their perceived deterrents in training (32–34).

### The implications and suggestions for improving rural specialists’ initial participation motivations

The present research found that different initial participating motivations had different effects on training effects. The educational preparation motivation played important roles in their learning engagement. Escape/stimulation motivation, on the contrary, may be in great need of intervention. Based on the previous researches and present findings, there are some suggestions for improving participation motivations.

First and foremost, government as well as medical and sanitary institutions at all levels should take effective measures to conduct vigorous propaganda about the necessity and importance of the GPs in society. Some information such as the status of general practice in developed countries, the social needs of general practice, and the bright future of GPs should be emphasized. At the same time, specialists should be provided much more information about the work of GPs, which can help specialists acquire comprehensive knowledge about future GPs.

Secondly, there should be inclinable policy to encourage specialists to become GPs. For example, the institution establishment of general practice at all levels should include supporting in financial budget, wage, treatment, and professional promotion for future rural GPs.

Thirdly, the base lines of specialists’ initial participation motivations to transition training towards future rural GPs should be measured with questionnaire or other approaches. If there are sufficient applicants, the selection of trainees should be necessary. However, if the quantities of applicants are not sufficient enough, the targeted intervention should be done according to the results of the base line measurement. In this manner, specialists’ inappropriate participation motivations, such as escape/stimulation, may transform into appropriate participation motivations, such as educational preparation.

Fourthly, more attention should be paid to the applicants with initial participation motivations of escape/stimulation. It is important to help them to objectively analyse both the advantages and disadvantages of participating in the transition training for future GPs. In particular, it is essential to help them realize that the development of a general practice system is a key point in the reform of national medical and health services. Specialists should take advantages of this opportunity to facilitate their professional development. In general, there is a great need to conduct sufficient communications to these specialists and turn their escape/stimulation motivations into educational preparation motivations.

In sum, the present research revealed the relationship among three variables, that is, initial participation motivations, perceived deterrents, and learning engagement in the training. Also, the findings of this research can provide suggestions for human resources departments in recruiting future rural GPs and help educators and administrators for effective training.

#### Strengths and limitations

The present research had some significant strengths. First, it investigated the predictive effects of specialists’ initial participation motivations and deterrents on their learning engagement in transition training for future rural GPs in China. Second, it found that specialists with escape/stimulation motivation perceived more deterrents and had lower learning engagement. Third, it revealed that the specialists with education preparation motivations had a strong predictive effect on their learning engagement in training, in spite of facing difficulties.

The limitations of this research were as follows: First, the participant samples were limited in number and scope, and the survey was limited to one province in mainland China. More participants should be investigated in the subsequent study. Second, the research method was limited and only questionnaire investigation was used. Third, this research only collected cross-sectional data and failed to gauge the longitudinal effects of participation motivations, deterrents, and learning engagement in transition training.

## Conclusions

Educational preparation motivations were the strongest predictors for learning engagement directly, while escape/stimulation motivations predicted learning engagement negatively through the full mediation of perceived deterrents in training. The findings have strong implications for recruiting and training efficacy in human resources for health. To facilitate specialists’ learning engagement in training, it is necessary to stimulate their initial education preparation motivations and reduce their escape/stimulation motivations to participation in transition training for future rural GPs.
